# Beyond the Scale: Setting Goals for Outcomes Beyond Weight in Evidence-based Obesity Treatment

**DOI:** 10.1007/s13679-026-00706-7

**Published:** 2026-03-30

**Authors:** Leah M. Schumacher, Jacqueline F. Hayes, KayLoni L. Olson, Samantha P. Flanagan, Doria Wohler, Katherine E. Darling

**Affiliations:** 1 Department of Social and Behavioral Sciences, Barnett College of Public Health, Temple University, Philadelphia, PA 19122 USA; 2 Center for Obesity Research and Education, Barnett College of Public Health, Temple University, Philadelphia, PA 19122 USA; 3 Weight Control and Diabetes Research Center, The Miriam Hospital, Providence, RI 02903 USA; 4 Department of Psychiatry and Human Behavior, Warren Alpert Medical School of Brown University, Providence, RI 02903 USA; 5 Clinical Family and Community Medicine, Lewis Katz School of Medicine at Temple University, Philadelphia, PA 19140 USA

**Keywords:** Obesity, Overweight, Goal setting, Patient-centered care, Patient-reported outcomes

## Abstract

**Purpose of Review:**

As definitions of obesity evolve beyond a sole focus on weight, evidence-based obesity treatments may also benefit from a broadening in focus. This review discusses benefits of setting goals with patients that expand beyond weight or anthropometric change, and reviews recent data on expected outcomes in four such domains where patients may often desire change: obesity-related diseases, health-related quality of life (HrQoL), body image, and weight bias internalization (WBI).

**Recent Findings:**

Lifestyle modification, obesity management medications, and metabolic and bariatric surgery can yield meaningful gains in obesity-related diseases, physical HrQoL, and body image. However, there is variability in response, and the expected magnitude of gains can differ by treatment approach and magnitude of weight loss. Less is known about WBI.

**Summary:**

Many patients are likely to see benefits beyond weight loss during treatment. Clarifying patients’ goals in these areas and aligning treatments accordingly can promote patient-centered care.

## Introduction

Recent definitions of obesity have moved beyond a sole focus on weight to recognize obesity as a chronic, progressive, relapsing, multi-factorial disease [1–5]. For instance, in 2025, the Lancet Commission on Diabetes and Endocrinology released a definition and diagnostic criteria for obesity that differentiates between preclinical obesity and clinical obesity and defines the latter as a chronic, systemic illness characterized by alterations in the function of tissues, organs, the entire individual, or a combination thereof, due to excess adiposity [1]. Other national and international working groups and organizations have released similar definitions [1–5]. While debate remains about whether, or under what circumstances, obesity is best considered a disease in its own right versus a harbinger of other diseases [1], these definitions highlight increasing recognition of obesity’s complex etiology, its potential direct impact on pathophysiology and health, and its chronic nature.

One impetus for developing improved diagnostic criteria has been mounting agreement around the limitations of a body mass index (BMI)-centric definition for obesity [1, 4]. Researchers, clinicians, scholars, and advocates alike have raised concerns about using BMI alone to accurately gauge current or future health risk on an individual level [6–8]. This concern has been paralleled by a growing chorus of individuals and organizations highlighting the very real and detrimental effects of weight stigma and bias [9–11]. As weight bias and stigma are often fueled at least in part by overly simplistic views of obesity that frame body weight as an attribute over which individuals have direct control [12, 13], concerns about bias and stigma further underscore the importance of accurately defining obesity.

Recent years have also witnessed an increased focus on holistic, person-centered obesity management [2, 14–16]. This is likely driven by many of the same factors as changes to the definition of obesity, as well as the expanded range of treatment options (e.g., newer obesity management medications [OMMs]), which offer more opportunities to tailor treatment to patients’ individual circumstances and preferences [14, 17, 18]. Additionally, there is significant heterogeneity in responses to evidence-based obesity treatments [19, 20], and weight loss maintenance remains challenging [21]. These factors further underscore the importance of using a personalized, holistic approach when working with patients. In sum, the field has recently been wrestling with many important questions about the nature, definition, and assessment of obesity.

**Fig. 1:**
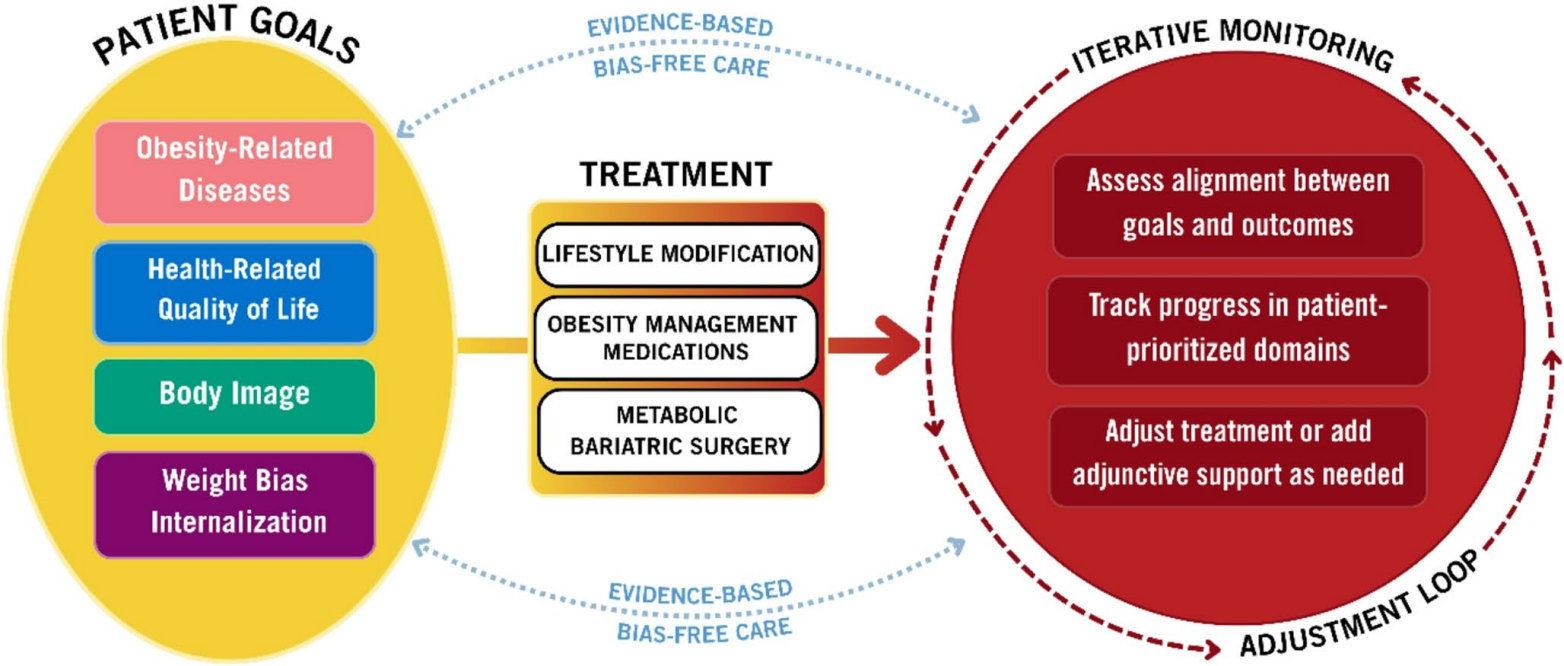
Potential domains for goal setting that extend beyond weight **. **The yellow oval on the left shows common domains in which patients may desire change and wish to set goals. The middle and right portions of the figure depict how information about these patient-prioritized domains may be used to set goals, iteratively monitor progress, and adjust plans as needed during evidence-based obesity intervention

With a more nuanced and comprehensive understanding of obesity, treatment approaches must similarly evolve beyond a sole focus on weight or BMI when defining “success” in obesity treatments [3, 22]. This review: (1) briefly discusses several potential benefits of broadening treatment goals beyond change in body weight, BMI, or adiposity when working with individuals pursuing evidence-based treatment approaches; and (2) reviews recent literature on the likely impact of evidence-based obesity treatments on four clinically meaningful domains in which patients may frequently desire change: obesity-related diseases, health-related quality of life (HrQoL), body image, and weight bias internalization (WBI; Fig. 1). We opted to focus on goal setting related to these domains since patients most often report seeking obesity treatment for health and/or appearance reasons [23–26]. Other domains, such as eating disorder symptomatology and mental health symptoms, may also be of interest to patients. While these may overlap with some topics reviewed herein (e.g., body image, HrQoL), a full review of expected impacts in these domains is beyond the scope of this review but is important for future research [27].

## Criticalness of Evidence-Based, Bias-Free Care

Before discussing goal setting for domains beyond weight, it is important to note that goals set with patients should be contextualized within the backdrop of evidence-based, bias- and stigma-free care. When patients express interest in obesity treatment, or when providers are considering recommending treatment, patients should be provided or referred to evidence-based approaches. At present, these are generally considered to be intensive lifestyle modification that involves ongoing support, OMMs, metabolic bariatric surgery (MBS), and, in some cases, endoscopic procedures [17, 28]. In most cases, advice to modify dietary intake or physical activity in the absence of other support (e.g., advice to simply “eat less and move more”) is insufficient to achieve clinically meaningful weight loss and should be avoided [29]. It is also imperative that providers proactively take steps to address and avoid weight bias and stigma. Considerable research has shown that weight bias and stigma can undermine treatment access and utilization [30] and have wide-reaching detrimental effects on physical and mental health [9, 31]. Consequently, it is essential that those working with patients understand what weight bias and stigma are, reflect on and challenge any biases they may hold, and actively work to create an inclusive care environment. Several consensus statements, reviews, and reports provide concrete strategies for doing this [4, 10, 32–34].

When thinking about high-quality obesity care, it is also important to distinguish between different types of goals that patients and providers may set. One type of goal is a behavioral goal (e.g., a goal to modify dietary intake). Behavioral goals can provide many benefits and are often part of evidence-based treatment programs. The merits of behavioral goals and effective strategies for setting them have been previously discussed [35–40]. Another type of goal setting involves identifying clinical or psychosocial outcomes that are meaningful to patients and for which they want to monitor their progress during treatment. The present review focuses on this latter person-centered goal setting (as opposed to behavioral goal setting). When selecting domains to focus on, shared decision-making and person-centered care principles may help guide conversations. These approaches are discussed in detail elsewhere [41, 42].

## Rationale for Setting Goals Beyond Weight, BMI, or Body Composition

Lifestyle modification interventions, OMMs, and MBS have historically—and are largely still—focused on change in weight or anthropometric characteristics as a primary metric of treatment success [43]. This is because improvements in anthropometric outcomes like weight are typically associated with a variety of benefits (see “Potential Outcomes of Interest Beyond Weight & Expected Treatment Impact” section below). In this spirit, the 2025 American Association of Clinical Endocrinology (AACE) Clinical Consensus Statement for obesity management in adults frames percent weight reduction as a “biomarker” that can be targeted to drive predictable improvements in health outcomes [3]. Others have similarly framed weight reduction as a surrogate marker of treatment effectiveness [28]. Despite weight being a viable treatment target, there are also potential downsides of focusing only or primarily on weight that argue for a broadening in focus.

First, focusing solely on weight may detract from the broader goal of improving health and well-being, which is why obesity treatment is typically considered by patients or recommended by healthcare providers [23, 24]. A specific number on the scale or a certain BMI has limited inherent value in and of itself [44]. Tangible gains in one’s health, functioning, and well-being that can result from weight and adiposity change are the ultimate goal for most patients and providers [3, 44]. Working with patients to set goals that extend beyond weight and anthropometric change can center the broader potential benefits of treatment.

Second, discussing goals beyond weight can help to promote alignment between patients’ motivations and expectations for treatment and the outcomes they are likely to experience. Patients may have a wide array of motivators for treatment [23, 45]. Different treatment modalities may be more or less likely to provide these desired benefits, as reviewed below. Proactively exploring patients’ reasons for treatment and then selecting a treatment approach and setting goals that match these motives can promote person-centered care and prevent alienating patients for whom weight loss is not a primary goal [3]. These conversations can also provide a valuable opportunity to assess and adjust expectations for treatment, as needed. For example, a patient may assume that weight loss will “fix” their body image concerns. However, this may or may not be the case. Fostering greater alignment between patients’ hopes for treatment and likely treatment outcomes may improve treatment adherence, patient satisfaction, and clinical outcomes [46, 47].

Lastly, having multiple metrics for evaluating progress can guard against a myopic and potentially unhelpful overfocus on weight and shape during treatment, both from patients and providers. In all treatment approaches, weight loss may stall at times [48, 49]. This can be due to many factors beyond the individual’s control (e.g., physiological adaptation [49]). Weight outcomes from obesity treatments also have large heterogeneity [19, 20, 50], and many patients may not reach their desired “goal weight” [18]. Selecting additional domains to focus on may help to buffer against potential frustrations that could otherwise derail treatment efforts, provide alternative and meaningful indicators of treatment success, and promote a more balanced relationship with one’s body [51].

## Potential Outcomes of Interest Beyond Weight & Expected Treatment Impact

Below, we review data on changes that patients may reasonably expect to experience during treatment in four areas pertaining to health and/or appearance—common reasons for seeking obesity treatment: obesity-related diseases, HrQoL, body image, and WBI. We also discuss pertinent clinical considerations related to these domains, such as how pre-existing concerns in these areas may impact treatment course.

### Obesity-Related Diseases

Obesity has pathophysiological overlap with or causally contributes to many diseases and disorders, including type 2 diabetes (T2D), cardiovascular disease, obstructive sleep apnea, osteoarthritis, and several types of cancer [1, 3]. Obesity can also lead to organ damage or dysfunction, contributing to adverse health events (e.g., heart attack) [1, 3]. To capture the diversity of obesity’s potential impacts on health, as well as the range of pathways underlying them, obesity-related diseases and complications are sometimes categorized as primarily cardiometabolic, biomechanical, or psychological in nature [3, 28]. Several reviews, clinical practice guidelines, and consensus statements discuss the health consequences of obesity and their mechanisms [1, 3, 28, 52]. From a goal-setting perspective, patients may hope to see risk reduction or clinical improvements in one or more of these conditions.

Robust evidence shows many obesity-related diseases and complications improve with obesity treatment, with a greater magnitude of weight loss generally relating to greater health gains [1, 3, 28, 53]. For some conditions, achievement of a clinically meaningful benchmark (e.g., > 5% or > 10%) may be necessary to provide benefits. The 2025 AACE Consensus Statement and other resources provide helpful graphics of expected health gains by amount of weight loss [3, 53]. These patterns highlight the importance of knowing what specific conditions patients most want to see gains in when selecting a treatment approach and setting outcomes for progress monitoring. Taking cardiovascular disease risk reduction as an example [54], the Look AHEAD trial, which tested an intensive lifestyle modification program against an education and support control among adults with overweight/obesity and T2D, observed many health benefits of intensive lifestyle intervention but no impact on cardiovascular disease outcomes [55, 56]. Mean weight loss in intensive lifestyle modification was < 10% [55]. In contrast, several studies of newer OMMs and MBS—which tend to produce weight losses > 10%—have shown benefits for cardiovascular disease outcomes [57, 58]. Additionally, post-hoc analyses from Look AHEAD revealed that patients who did lose > 10% experienced cardiovascular risk reduction [59]. Thus, if cardiovascular disease is a primary concern for patients, a treatment approach that yields greater weight loss may be most appropriate.

While weight loss magnitude commonly parallels the degree of expected improvements in obesity-related diseases, growing evidence suggests treatments may also have weight-independent effects. This can be seen in data emerging from trials of newer OMMs [57, 60, 61], as well as the MBS literature [62]. For instance, some of the cardiovascular benefits of semaglutide use appear to be due to yet unconfirmed weight-independent mechanisms [57]. Similarly, MBS improves glucose metabolism through weight-dependent and weight-independent mechanisms (e.g., improved hepatic insulin sensitivity [62]). Even in lifestyle modification trials, factors like nutrition and cardiorespiratory fitness—which are also targeted in treatment, though often in relation to weight loss—can differentially affect body composition and health improvements [63, 64]. These data underscore the importance of looking at factors beyond weight loss when gauging treatment benefit, as well as the importance of considering the full benefit profile of each treatment option and its alignment with patients’ goals when selecting a treatment approach.

### Health-Related Quality of Life

HrQoL refers to an individual’s perception of their health and how it impacts their daily life [65]. This is often a meaningful patient-centered goal to measure and monitor during treatment [66]. Although definitions and subcategories vary, HRQOL can encompass two broad domains: physical and psychosocial. Physical HRQoL pertains to an individual’s perceived well-being as influenced by physical health, illness, and injury. Common subcategories include physical functioning in daily activities, bodily pain or discomfort, and role limitations based on physical health problems [67, 68]. Psychosocial HRQoL pertains to an individual’s social and emotional well-being. Common subcategories include mental health, social functioning, and role limitations based on emotional well-being [67, 68]. Several validated questionnaires can be used to measure HrQoL (see Table 1), including measures specific to weight, which may allow for a more sensitive measure of how excess adiposity impacts HrQoL.

**Table 1:** Validated, self-report measures of potential non-weight outcomes of interest

Measure Name	Brief Description
Health-Related Quality of Life (HrQoL) Measures	
Short-Form 36 (SF-36) [67]	Widely used, 36-item measure of general HrQoL with 8 subscales.
EuroQOL [68]	25-item measurement of general HrQoL encompassing five dimensions.
WHOQOL-BREF [123]	26-item measure of general HrQoL addressing 4 domains.
Impact of Weight on Quality of Life-Lite (IWQOL-Lite) [124]	31-item measure of obesity-specific quality of life in adults. A briefer version specific to concerns in obesity clinical trials is also available [114].
BODY-Q [131]	Questionnaire with multiple, independently functioning scales that evaluate body-specific concerns, including a subscale of HrQoL specific to obesity.
Body Image Measures	
Eating Disorders Examination-Questionnaire [126]	28-item measure of eating disorder psychopathology. Relevant subscales include shape concern and weight concern.
Body Shape Questionnaire [127]	Self-report measure of preoccupation with body shape. Original version is 34 items, but 8- and 16-item versions are available. [128]
Body Appreciation Scale-2 [132]	10-item measure of positive body image.
Internalized Weight Bias Measures	
Weight Bias Internalization Scale – Modified [133]	11-item updated version of the Weight Bias Internalization scale.
Weight Self Stigma Questionnaire [129]	12-item measure including dimensions of self-devaluation and fear of enacted stigma intended for individuals with overweight or obesity.
Anti-Fat Attitudes Questionnaire [134]	13-item survey with three subscales of dislike, fear of fat, and willpower.
Attitudes Towards Obese People [130]	20-item questionnaire measuring stereotypical attitudes towards individuals with obesity.
Beliefs About Obese People [130]	8-item survey measuring an individual’s beliefs about the causes and controllability of obesity.

Physical HrQoL in individuals with obesity is frequently poorer when compared to individuals without obesity, and it tends to decrease as weight increases [65]. For instance, compared to individuals with a non-overweight BMI, individuals with obesity and severe obesity had 1.21 and 1.87 greater odds ratios of reporting greater than 14 unhealthy days in the prior month [69]. This is perhaps unsurprising given obesity’s association with many physical health conditions and its potential to make activities of daily living more difficult or painful (e.g., due to mobility issues). Physical HrQoL consistently improves with obesity treatment; this is true across treatment modalities [70, 71]. Improvements are primarily driven by weight change and show a positive association with weight loss magnitude [65, 70, 71]. Given this relationship, medical interventions such as MBS typically result in larger improvements in physical HRQoL compared to lifestyle interventions [65]. For instance, a subset of individuals from the Longitudinal Assessment of Bariatric Surgery trial showed increases of 6.7–13.5 in the physical component subscale of the SF-36 over 1 year [72], whereas lifestyle intervention using the Diabetes Prevention Program showed improvements of 1.4 points relative to control over 1 year [73]. Medication trials of GLP-1s have also shown consistent improvement in physical HrQoL, with approximately 15% more participants achieving clinically meaningful improvement compared to the placebo group [74]. Improvements in physical HrQoL are typically largest in the period immediately following intervention, then decline over time [65]. Moreover, individuals with lower baseline functioning tend to see greater improvements [75]. Other mechanisms beyond weight impacting physical HrQoL may include greater physical activity [76, 77], which can lead to improvements in cardiorespiratory fitness, muscle strength, and mobility, and improvements in diet quality [78], which can reduce inflammation, address nutrient deficiencies that impact physical HrQoL, and improve energy.

Similar to physical HrQoL, psychosocial HrQoL tends to be poorer in individuals with obesity compared to those without, particularly as obesity severity increases [79, 80]. For instance, compared to individuals with a non-overweight BMI, individuals with obesity and severe obesity had 1.17 and 1.41 greater odds ratios of reporting greater than 14 unhealthy mental health days in the prior month [69]. This relationship is likely a product of a complex interplay of social, psychological, and biological factors [81, 82]. Psychosocial HrQoL often improves following obesity treatment, though outcomes tend to be more variable, with some meta-analyses indicating improvement and others not [27, 70]. Findings implicating the role of weight loss in affecting psychosocial improvement are also mixed [70]. As with physical HrQoL, lifestyle changes—independent of weight loss—can positively influence psychosocial HrQoL [76–78]. For instance, higher levels of physical activity are associated with improved mood, lower stress, better cognitive function, and better sleep habits that may positively impact psychosocial functioning [83–85]. Skills commonly taught in lifestyle intervention programs (e.g., problem-solving) and treatment elements like social support and a sense of belonging (for group-based interventions) may also impact psychosocial HrQoL [86–88]. Children, in particular, may benefit from the non-weight-related effects of treatment, as evidenced by data showing improvements in psychosocial HrQoL during lifestyle interventions that are independent of weight change [89–91].

### Body Image

Negative body image is common among individuals with higher body weight [92], impacting 50–70% of adults [93]. Body image is a broad and multifaceted concept, with substantial literature linking it to body weight, weight-related behaviors, and overall well-being [94]. For example, individuals with obesity report greater *body dissatisfaction*, a subjective negative evaluation of one’s body and/or parts of the body, compared to individuals with lower body weight [95]. Individuals with higher body weight are also at risk for *overvaluation of weight and shape*, which occurs when negative feelings about body weight and/or shape are a strong influence on an individual’s overall sense of self-worth [96]. Overvaluation of weight and shape is less common than body dissatisfaction, affecting 20% of individuals seeking obesity treatment [96], but tends to be more enduring and is associated with increased risk for maladaptive weight-related behaviors and psychosocial distress [97]. Negative body image is generally more prevalent among women, younger individuals, and those seeking obesity treatment [95, 98–100]. Across the body weight spectrum, the harmful consequences of negative body image are well documented, leading some to argue it is a largely overlooked public health concern [101].

Body image can play a central role in initiating weight loss efforts across treatment modalities [102–105]. It may also be a valuable outcome to monitor during treatment. Weight loss is often associated with improvements in several features of body image (e.g., body appreciation, body dissatisfaction, shape concerns) [106, 107]. As a result, poor body image is conceptualized as a common consequence of high body weight and weight loss is viewed as a practical therapeutic approach [108]. However, the benefits of obesity treatment for body image can be complicated. Only two-thirds of adults achieve clinically meaningful weight loss during gold-standard treatment and weight regain is expected [109]. Further, there is evidence of a “legacy” effect, such that negative body image features persist even after weight loss, albeit attenuated [108]. Among those with significant weight loss, new body image concerns can emerge following treatment (e.g., excess skin) [110].

Body image can also disrupt weight loss efforts. Several studies have found that individuals who attempt weight loss primarily for appearance-related reasons are at risk for discontinuing treatment, achieving less weight loss, and poorer weight maintenance [25, 26, 105, 111]. Further, individuals who enter treatment with greater body shape concerns are less likely to adhere to weight loss recommendations [112, 113].

Taken together, the existing literature highlights the potential clinical benefit of body image assessment and monitoring. Assessing appearance-related motivation for weight loss along with key features of body image (e.g., body dissatisfaction, overvaluation of weight and shape) can help inform treatment decisions and identify risk for difficulty fully engaging in treatment. Additionally, if patients are seeking improvements in body image but continue to struggle with these concerns, they can be referred to adjunctive methods for body image improvement (e.g., regular exercise, cognitive behavioral therapy, dissonance-based treatments that target societal beauty ideals and pressures).

### Weight Bias Internalization

WBI, defined as the application of negative weight-related attributions to oneself and self-devaluation because of weight, is common among individuals with obesity, including those seeking obesity treatment [12, 114]. Exposure to societal weight stigma—i.e., social rejection, devaluation, or discrimination of an individual based on their weight status [13]—often contributes to WBI. Meta-analysis has found that high WBI is concurrently associated with poorer psychosocial (e.g., mental health, self-compassion, social functioning), physical (e.g., weight status, quality of life, general physical health), and behavioral health (e.g., disordered eating, physical activity, healthy eating) outcomes, as well as healthcare avoidance [11, 30, 31]. Further, in lifestyle modification programs, higher baseline WBI scores have been associated with a 37% reduction in likelihood of achieving a ≥ 5% weight loss and 34% reduction in achieving ≥ 10% weight loss at 24 weeks [102], and patients with the lowest WBI scores at baseline have lost almost twice as much weight as patients with the highest WBI scores [115]. Data on the bidirectional association between changes in WBI and weight status over treatment are limited. Some research has shown a significant decrease in WBI during the course of lifestyle modification [115] and after MBS [116, 117], while other research has found that weight change does not predict change in WBI [102]. More longitudinal data is needed to understand whether and for whom WBI naturally improves during treatment and the long-term sustainability of improvements.

As noted above, many organizations have released consensus statements about the importance of addressing weight stigma and WBI [10, 32, 33]. Several of these explicitly recommend including assessment and experience of weight stigma and WBI in the staging of obesity severity, as well as addressing these issues in treatment [33, 118]. However, limited guidance is provided on how to do this [119]. Literature on how WBI can best be addressed through intervention is only emerging. However, prior work has identified multiple potential benefits of addressing internalized weight bias and self-devaluation [11, 120]. For example, one small self-compassion intervention observed improvements in WBI, body shame/surveillance, and self-compassion [121]. Other studies have identified preliminary effectiveness of WBI interventions, both independently and alongside obesity treatment [11, 120]. As researchers continue to identify and test ways to reduce WBI, approaching weight management in a patient-centered way, with attention towards WBI and its potential effects on patient well-being and treatment progress, will likely prove critical.

## Discussion

While definitions of obesity have recently evolved, most obesity treatment studies—and, likely, many interactions in clinical practice—continue to focus largely on weight loss [43, 122]. This review builds on other calls for a more “complications-centric” approach to obesity management [3] by discussing the recent literature on several domains beyond weight in which patients may wish to set goals during treatment. To our knowledge, it is the first to include a comprehensive discussion of treatment effects on body image and WBI, important considerations for many patients.

In general, data show that evidence-based obesity treatments produce improvements in multiple physical health conditions associated with obesity, physical HrQoL, and several facets of body image [3, 28, 53, 65, 106, 107]. This is encouraging, as it suggests that many patients may benefit from monitoring their progress in these areas and seeing tangible gains with treatment. However, as with weight loss, outcomes in these domains can be heterogeneous, improvements may not always be linear, and not all patients may experience benefits. This underscores the importance of tracking patients’ individual progress in these areas, if important to them, and connecting patients to additional resources to address unresolved concerns as needed. Because setbacks and heterogeneity in treatment responses are common and to be expected, it is also important to address these issues clinically and not frame them as patient failure to avoid stigmatizing or blaming patients.

Additionally, different treatment modalities may have differential effects. For example, lifestyle modification may yield meaningful improvements in physical HrQoL and body image, but have less impact on certain obesity-related diseases. Conversely, approaches that produce greater mean weight loss (i.e., MBS, newer OMMs) may yield greater physical health and HrQoL gains, but also increase risk of emergent body image concerns (e.g., due to excess skin) [110]. Understanding patients’ primary goals for treatment beyond weight can help ensure patients are matched with an approach likely to provide these benefits.

Given the data reviewed here as well as broader advocacy for a re-focusing in obesity treatment from weight loss per se to health and functioning [3], we propose a model (Fig. 1) in which patients’ broader concerns and motivators for treatment are routinely assessed at treatment onset, factored into initial treatment decisions, and then regularly monitored during treatment, with data feeding back to inform obesity treatment adjustments and use of additional supports/approaches (e.g., counseling). Clinically, providers may find it helpful to use validated self-report measures to assess and monitor patients’ progress with HrQoL, body image, and WBI. Table 1 provides a list of measures that can be used for these purposes [67, 68, 123–134]. Data obtained from these measures can be used in clinical conversations, much like laboratory results might be, to determine the best path for care, when considered alongside other data sources (e.g., patient satisfaction, health improvements, weight or adiposity change). Given time constraints in many clinical settings [135], some patients, especially those with more severe or enduring concerns, may benefit from dedicated psychological or behavioral health support related to these concerns [16].

There are several important directions for future work in this area. While efforts are underway to develop obesity therapeutic targets beyond mean percent weight loss that reliably predict optimal physical health gains [22], research is needed to determine what outcomes (e.g., body composition change, improved quality of life) are best to orient patients toward, and how to implement this in clinical practice. Further, much remains to be learned about WBI and its reduction [11]. Similarly, there is a need for more body image research in diverse, historically underrepresented populations. The last 15 years have seen significant growth in the study of positive features of body image (e.g., body appreciation, appearance satisfaction), which hold promise for buffering against the harms of societal body-related pressures and their impact on health and well-being [136]. There is also a great need and opportunity for payers and policymakers to expand metrics of obesity treatment “success” [122]. At present, for example, patients may lose coverage for OMMs if they are not reaching certain weight loss targets, regardless of whether they are experiencing other gains. Many health systems may also require close monitoring of weight and use weight as a primary metric for dictating treatment course (e.g., eligibility for specialty services). Continued study of the broad benefits of obesity treatments, including those independent of weight change, and of the patient-reported outcomes most important to patients may help shift such policies.

## Conclusion

As obesity is increasingly recognized as a complex chronic disease, it is increasingly clear that treatment success should focus on more than weight change alone. Broadening clinical focus to include outcomes like obesity-related diseases, HrQoL, body image, and WBI offers a more accurate reflection of patients’ lived experiences and can foster more patient-centered, personalized care [137]. This paper provides a comprehensive review of the expected effects of obesity treatments on these domains and advocates for the routine assessment, monitoring, and use of data on these outcomes to inform care in clinical settings. Practical suggestions for ways to approach evaluation of and treatment planning in light of this information are provided. Continued research on non-weight treatment targets and shifts in payer and policy structures to recognize multifaceted definitions of treatment “success” will also be critical to aligning obesity care with contemporary scientific understanding and patient-centered values.

## Key References

- D’Adamo L, Shonrock AT, Monocello L, Goldberg J, Yaeger LH, Pearl RL et al. Psychological interventions for internalized weight stigma: a systematic scoping review of feasibility, acceptability, and preliminary efficacy. J Eat Disord. 2024;12(1):197. 10.1186/s40337-024-01132-7
  - ◌ Systematic scoping review of 20 studies examining psychological interventions for internalized weight stigma (IWS). Studies generally demonstrated high feasibility, acceptability, and engagement. Sixteen of the twenty studies observed significant reductions in IWS maintained over follow-up periods; however, results comparing intervention versus control conditions were mixed. Discrepancies between measurement tools were also noted.
- Dijkhorst PJ, de Vries CE, Terwee CB, Janssen IM, Liem RS, van Wagensveld BA, et al. A Core set of patient‐reported outcome measures to measure quality of life in obesity treatment research. Obes Rev. 2025;26(2):e13849.
  - ◌ Study reporting results from the second and third global Standardizing Quality of Life in Obesity Treatment consensus meetings. Emphasizes the lack of standardization in patient-reported outcome measures (PROMs) that has made it difficult to adequately gauge and compare quality of life outcomes in research focused on obesity treatment. Selected nine patient-reported outcomes and three PROMs to serve as a “core set” for future obesity treatment research
- Flølo TN, Liu HH, Andersen JR, Kolotkin RL. Exploring the relationship between obesity, weight loss and health‐related quality of life: An updated systematic review of reviews. Clin Obes. 2020:e70049.
  - ◌ Systematic literature review (SLR) of reviews that updated a previous SLR by examining data from eight SLRs and/or meta-analyses published since 2017 focused on the impact of obesity and weight loss on health-related quality of life (HRQoL). Results consistently demonstrated a negative association between obesity and HRQoL, with some evidence suggesting poorer HRQoL in people with adverse metabolic profiles. Also found substantial weight or BMI reduction related to significant and clinically relevant HRQoL improvements, with pronounced and persistent effects on the physical components in particular.
- Nadolsky K, Garvey WT, Agarwal M, Bonnecaze A, Burguera B, Chaplin MD, et al. American Association of Clinical Endocrinology consensus statement: Algorithm for the evaluation and treatment of adults with obesity/adiposity-based chronic disease–2025 update. Endocr Prac. 2025;31(11):351-1394.
  - ◌ Updated obesity algorithm that emphasizes topics such as a complications-centric approach to obesity management and the importance of person-centered care. Encourages use of the comprehensive diagnostic term Adiposity-Based Chronic Disease (ABCD) to encompass all aspects of obesity as a chronic disease.
- Pearl RL. Internalization of weight bias and stigma: Scientific challenges and opportunities. Am Psychol. 2024;79(9):1308.
  - ◌ Comprehensive review article that synthesizes current knowledge on weight bias internalization, examining its conceptualization, measurement, prevalence, correlates, and health implications. Also discusses critical scientific challenges, including measurement complexities, minimal empirical research, and mechanisms of action.
- Rubino F, Cummings DE, Eckel RH, Cohen RV, Wilding JP, et al. Definition and diagnostic criteria of clinical obesity. Lancet Diabetes Endocrinol. 2025;13(3):221-62.
  - ◌ Report produced by a Commission of 58 global experts that provides an updated definition and diagnostic criteria for obesity. Differentiates between preclinical and clinical obesity and proposes a two-tiered diagnostic approach, which requires confirmation of excess adiposity through anthropometric measures beyond BMI or direct body fat measurement, followed by assessment against 18 diagnostic criteria for clinical obesity based on organ dysfunction or functional impairment.
- Sherifali D, Racey M, Fitzpatrick‐Lewis D, Greenway M, Sockalingam S, Wada J, et al. Missing the target: A scoping review of the use of percent weight loss for obesity management. Obes Rev. 2025:e13960.
  - ◌ Scoping review with 30 studies examining how percentage weight loss targets have been used in obesity management research. Results revealed most studies targeted 3-10% body weight loss, which was not feasible or sustainable for many participants. Discusses the potential value of more comprehensive, patient-focused parameters—includes those co-developed with patients—as treatment targets.

## Data Availability

No datasets were generated or analysed during the current study.
